# Fundamental Study for a Graphite-Based Microelectromechanical System

**DOI:** 10.3390/mi9020064

**Published:** 2018-02-02

**Authors:** Junji Sone, Mutsuaki Murakami, Atsushi Tatami

**Affiliations:** 1 Faculty of Engineering, Tokyo Polytechnic University, Atsugi 243-0297, Japan; 2 Material Solutions Research Institute, KANEKA Corporation, Torikai-Nishi 5-1-1, Settsu, Osaka 566-0072, Japan; Mutsuaki.Murakami@kaneka.co.jp (M.M.); Atsushi.Tatami@kaneka.co.jp (A.T.)

**Keywords:** carbon-MEMS, cantilever, doubly clamped beam, resonance frequency, HOPG, graphite sheet

## Abstract

We aimed to develop a process for constructing a carbon-based microelectromechanical system (MEMS). First, we prepared a highly oriented pyrolytic graphite (HOPG) crystal microsheet by exfoliation. We fabricated cantilevers and a double-clamped beam by controlling the thickness of the HOPG microsheet using a MEMS process. Second, we used a graphite sheet with contour line adhesion by metal sputter deposition. Third, we used a highly accurate graphite sheet with face adhesion and laser cutting. The first resonance frequencies were evaluated. We confirmed improvement in *Q* values to 1/10 level of a quarts vibrator, high performance, and a simple structure.

## 1. Introduction

Carbon nanotubes, fullerenes, and graphene are materials are useful in the fabrication of electrical devices (e.g., transistors, electrical wires, and batteries), microelectromechanical systems (MEMS) [1], and nanomechanical technologies [2]. These materials have high mechanical strength, high electrical mobility, chemical stability, and high thermal conductivity.

One method, a freestanding, high-carbon structure for MEMS devices [3,4], was fabricated by using glassy carbon. Molding and soft lithography were performed to obtain structures with a smooth shape. The carbon used was pyrolyzed from polymeric precursors. The carbon-based materials exhibited a porous microstructure, shrinkage deformation, and a pyrolysis temperature that was slightly higher than that required for a MEMS device. Dinh et al. developed graphite drawn on polyvinylchloride (GoPo) and graphite-on-paper devices [5,6]. The resulting devices are effective as wearable sensors. Our target is MEMS and more highly sustainable devices that function under severe conditions.

For nanoelectromechanical systems (NEMS), many studies have been done on highly oriented pyrolytic graphite (HOPG) crystal using graphene that can undergo multiple exfoliations. Rose et al. [7] fabricated a cantilever through the direct mechanical cleavage of a bulk HOPG crystal on silicon micropillars by using a focused ion beam, which requires micropillars. Chen et al. [8] also fabricated monolayer doubly clamped beams by using monolayer graphene flakes. A resonance frequency was observed to be near 65 MHz. Cantilevers and doubly clamped beams with thicknesses varying from a single atomic layer to 75 nm were fabricated. Bunch et al. [9] observed that resonance frequencies varied from 1 MHz to 170 MHz. The aforementioned studies, however, require sophisticated fabrication techniques, such as electron beam lithography.

The primary goal of our research was to develop a microelectromechanical system (MEMS) that exhibits superior mechanical properties for increasing industrial applications. Therefore, we used MEMS fabrication and integrated a carbon structure. First, we attempted to fabricate cantilevers using a conventional microelectromechanical system (MEMS) fabrication technology [10,11] and highly oriented pyrolytic graphite (HOPG) [12]. However, we could not fabricate high-performance devices because 10-μm cracks generated from peeling and carbonizing the metal caused variation in the character of the graphite. In addition, the mechanical strength of HOPG is one-third of that of graphene. Therefore, utilizing graphene to maximize the performance of a MEMS would require an improvement in graphite sheets. We next used a highly accurate graphite sheet to fabricate vibrators while avoiding the generation of 10-μm cracks. The graphite sheet was bonded along a contour line to the metal base of a MEMS structure using sputter deposition. To achieve better performance, we used a highly accurate graphite sheet and used face adhesion to bond the graphite sheet. The vibrator shape was produced by laser cutting. The quality of the vibrators was assessed by measuring the first resonance frequency.

## 2. Resonance Frequency of a Graphite Sheet

Graphite is an anisotropic material. Kelly considered the stress and strain relation of a hexagonal lattice, such as graphite [13]. Both the elastic modulus and compliance can be experimentally measured using acoustic wave propagation, ultrasonic testing, flexural vibrations, and static stress–strain measurements. Solving these equations, Kelly determined that the Young’s moduli parallel and perpendicular to the basal plane are 1/*S*_11_ and 1/*S*_33_ (where *S* is the compliance), respectively. The following equation has been derived for the first resonance frequency for a bending vibration:

(1) $$f=A\frac{t}{L^{2}}\;\sqrt{\frac{E}{\rho_{0}}}$$

where *E* = 1/*S*_11_, *ρ*_0_ is the mass density (2200 kg/m^3^), *t* and *L* are the thickness and length of the suspended graphene sheet, respectively, and *A* is equal to 1.03 for doubly clamped beams and 0.162 for cantilevers.

Bunch measured 33 resonators with thicknesses varying from a single atomic layer to 75 nm [14]. They fitted the results using the resonant equation with *E*. The results show the relation between frequency and *t*/*L*^2^ for multilayer graphene, plotted for the frequency range corresponding to calculated *E* values between 1.0 and 2.0 TPa. We used *E* = 1.0 TPa for high-accuracy graphite and *E* = 0.3 TPa for HOPG on the basis of the properties reported by Lu [15]. In our method, *Q*-values are calculated by the 3 dB method.

## 3. MEMS Fabrication Process Using HOPG

### 3.1. Fabrication Method

**Figure 1:**
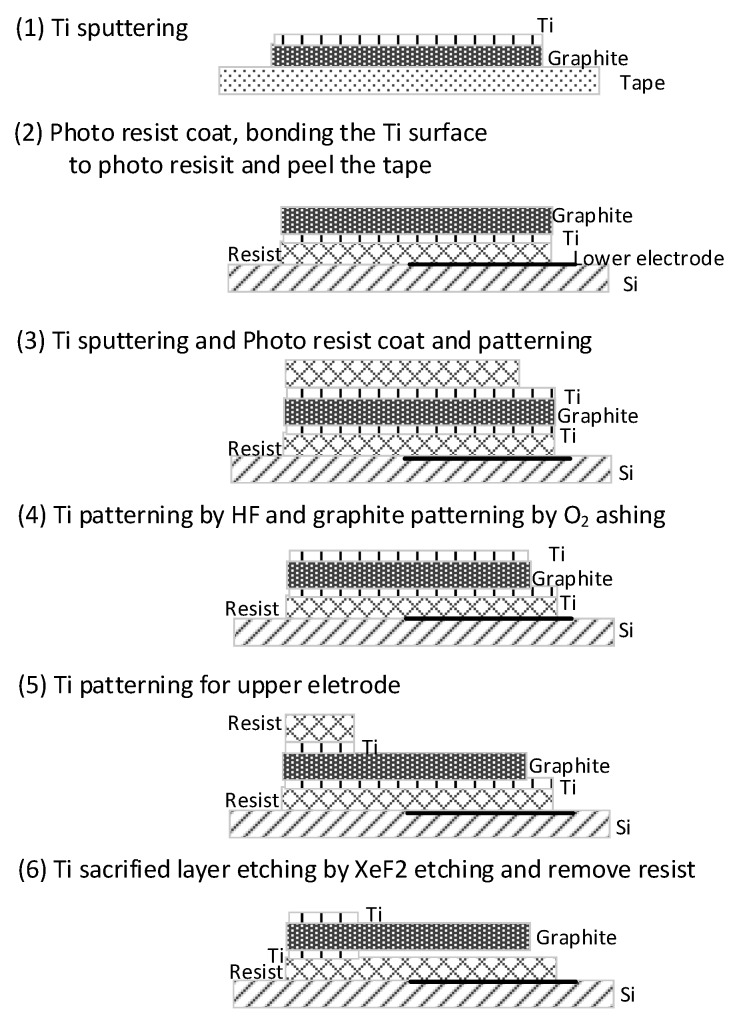
Scheme of fabrication process.

Our aim was to fabricate a complex device consisting of micro- and nanoelectromechanical systems using a typical MEMS fabrication process. The authors used the exfoliation method to prepare HOPG microsheets, as other chemical methods cannot produce 1-µm-thick films for MEMS devices. The initial stage of the fabrication process (Figure 1) is as follows: the HOPG microsheet was exfoliated almost 10 times using adhesive tape to produce an approximately 3-µm-thick HOPG sheet. (1) A tantalum coating (40 nm) was applied by sputtering one side of the sheet; (2) The HOPG sheet with its tantalum coat was attached to a Si plate (patterned lower electrode) by using a resist and a hot plate. In the second stage, the HOPG microsheet was polished, and its thickness was reduced to almost 1.0–0.5 µm on soft rubber by using wet sandpaper numbers 2000, 3000, and 6000. The resulting surface roughness was almost 0.1 µm; (3) A tantalum coating was applied by sputtering the side of the sheet. The cantilever pattern was formed by using a resist; (4) The upper tantalum coat (outside pattern) was subjected to wet etching. The HOPG microsheet (outside pattern) and resist were removed by O_2_ ashing; and (5) The upper electrode was formed by resist patterning. The tantalum film of the outside pattern and the cantilever underneath were removed by XeF_2_ dry etching [16], which is the appropriate dry-etching technique for SiO_2_ and Si. The etching rate of the tantalum layer was almost 250 nm/min for an undercut. Therefore, in the case of a 10,000-nm beam width, etching of the sacrificial layer requires 40–50 min. The tantalum layer of the outside pattern will be used as an upper electrode; in this experiment, we did not generate the upper electrode by patterning (5) and lower electrode patterning.

In our first trial, we directly used an exfoliated HOPG microsheet with a thickness of more than 2 µm and surface roughness of 0.5 µm. Such a thickness requires a long fabrication process, which leads to damage of the HOPG microsheet, because it takes 50 min for completion of O_2_ ashing. Therefore, the cantilever was non-functional.

**Figure 2:**
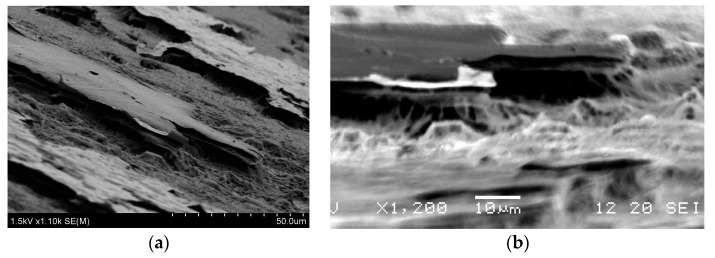
SEM image of over all of the C5 cantilever and clearance. (**a**) Cantilever: C5; (**b**) Clearance of cantilever.

In our second trial, we polished and exfoliated the HOPG microsheet to allow the cantilever to function and the fabrication process for the doubly clamped beam. We used wet sandpaper numbers 2000, 3000, and 6000 to reduce the thickness of the microsheet to around 500 nm. Figure 2 shows a SEM image of the cantilever after O_2_ ashing. A uniform HOPG microsheet was formed because of the polishing of the exfoliated HOPG microsheet. Our fabricated MEMS device exhibits rounding at corners and numerous cracks, making a precise measurement of the thickness difficult. We observed the device at a 60° tilt using scanning electron microscopy (SEM, JSM-5600, JEOL Ltd., Tokyo, Japan). The thicknesses at the round part, thick part, and cracks differ. We estimated the thickness at the thinnest part. Photoresists are damaged by O_2_ ashing at one corner. The SEM micrographs of the underside of the devices lack detail because of the low resolution of the instrument.

### 3.2. Fabrication Results

**Figure 3:**
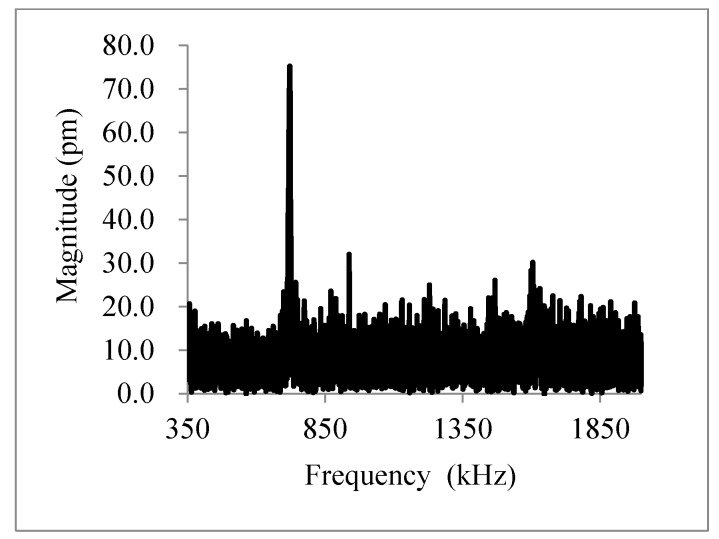
Resonance frequency of cantilever: C5.

**Figure 4:**
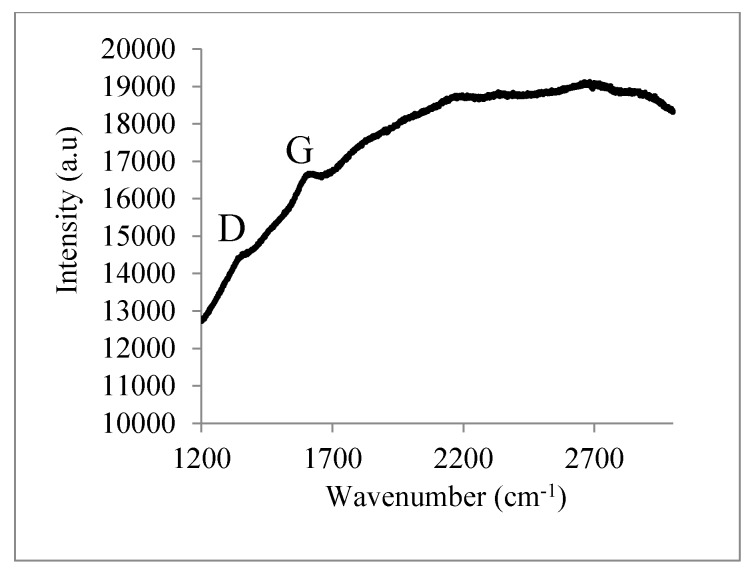
Raman spectra of the cantilever: C5.

**Figure 5:**
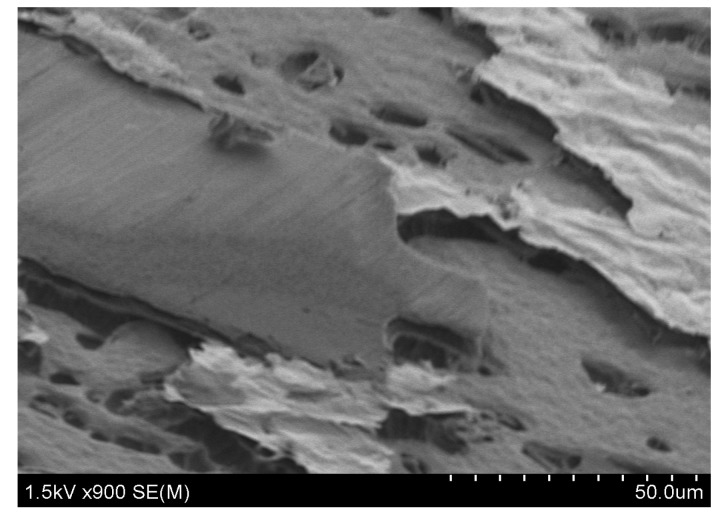
SEM image of the C11 cantilever.

**Figure 6:**
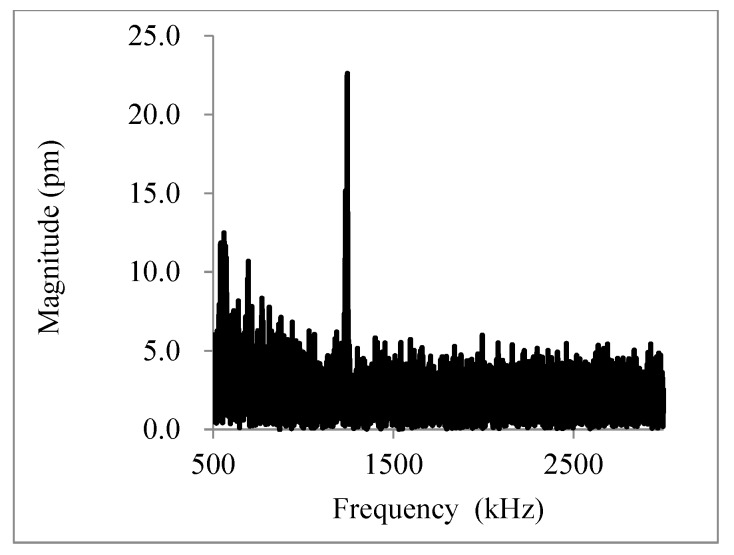
Resonance frequency of cantilever: C11.

**Figure 7:**
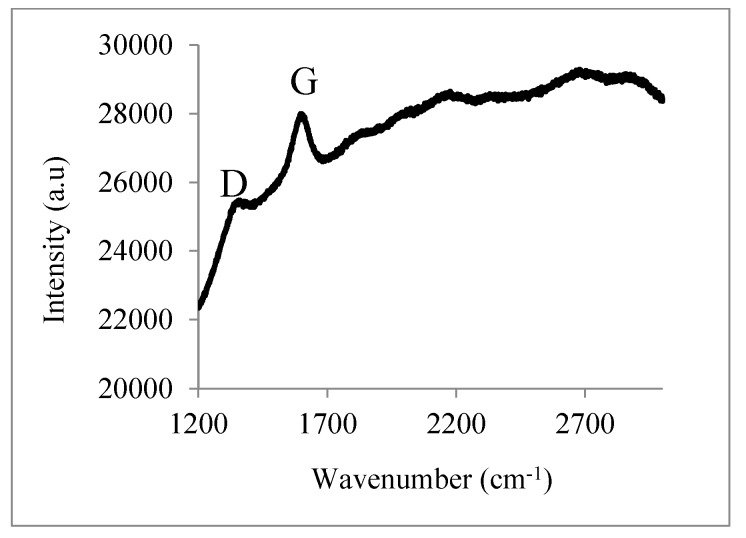
Raman spectra of the cantilever: C11.

Figure 2 shows the fabricated cantilever and the clearance between the HOPG microsheet and resist layers. In this case, the clearance was almost 40 nm. High-resolution observation was difficult because the electron beam damages carbon. Figure 3 displays the results of the frequency measurement. We used an MSA-500 microsystem analyzer (Polytec Inc., Waldbronn, Germany). We used a 10× microscopic lens and a laser Doppler vibrometry scanner (built in MSA-500) and device on a piezoelectric vibrator film fixed by double-stick tape. The device was placed in the vacuum chamber, which was evacuated to approximately 10 Pa by a dry scroll pump. With this setup, we could measure resonance frequencies as high as 20 MHz. We inferred that the fixing method would generate degeneracy for the high-frequency measurements. We confirmed by Raman spectroscopy (NRS-3100, JASCO Co., Tokyo, Japan) that the HOPG microsheet was on the surface of the cantilever (Figure 4). Figure 5, Figure 6 and Figure 7, respectively, show an image of another fabricated cantilever, the results of the frequency measurement, and Raman spectra of the cantilever. We observed peaks for G and D bands in the Raman spectra.

Table 1 shows the theoretical and measured resonance frequencies of the cantilever. The first resonance frequency *f* is given by Equation (1). We used an *E* value of 300 GPa (HOPG) in the computations.

**Figure 8:**
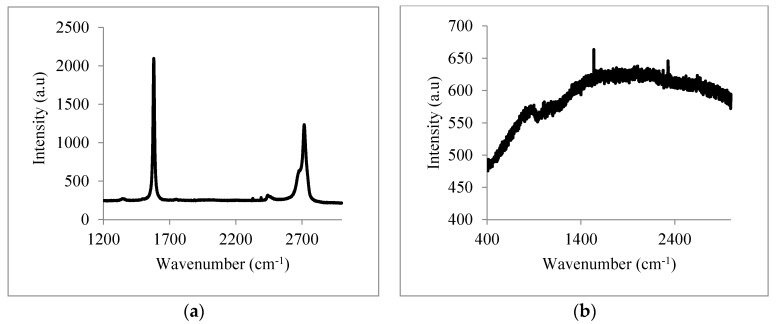
Raman spectra of the highly oriented pyrolytic graphite (HOPG) film and tantalum film. (**a**) HOPG film; (**b**) Tantalum film.

The ratio of the measured frequency to the computed resonance frequency is approximately 0.28–1.018. Figure 2 and Figure 5 show that C5 had a thick region and a thickness that was more uniform than that of C11. Thus, the ratio of the measured frequency to the calculated resonance frequency of C5 was slightly higher than that of C11. The resonance frequency was probably affected by the nonuniform surface thickness and the numerous cracks in C5. Figure 8 shows the Raman spectra of the HOPG microsheet and of the tantalum film formed by sputtering. Spectra in Figure 4 and Figure 7 also suggest defects (D band, peak at around 1400 cm^−1^) and the effect of tantalum. The property of the HOPG microsheet declined relative to the original value. One main effect was O_2_ ashing, which is a plasma-based process. The tantalum film was removed by hydrofluoric acid (HF) etching. Because of the strong adhesion of tantalum to carbon, it was difficult to completely remove the tantalum from the surface of the cantilever.

**Figure 9:**
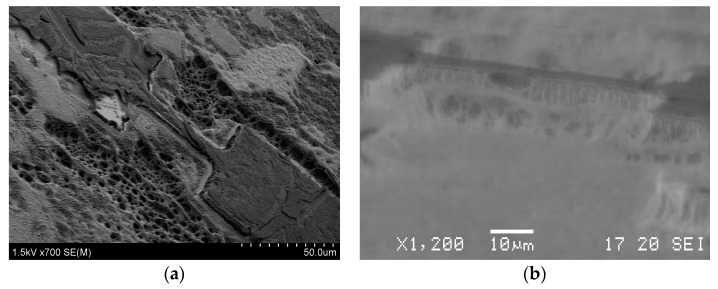
SEM image of over all of the DB501 doubly clamped beam and clearance. (**a**) Doubly clamped beam: DB501; (**b**) Clearance of doubly clamped beam.

**Figure 10:**
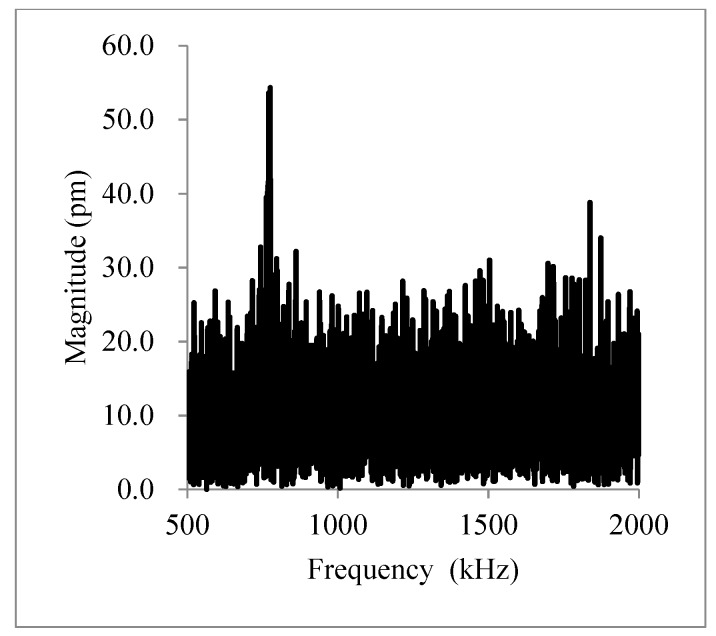
Resonance frequency of doubly clamped beam: DB501.

**Figure 11:**
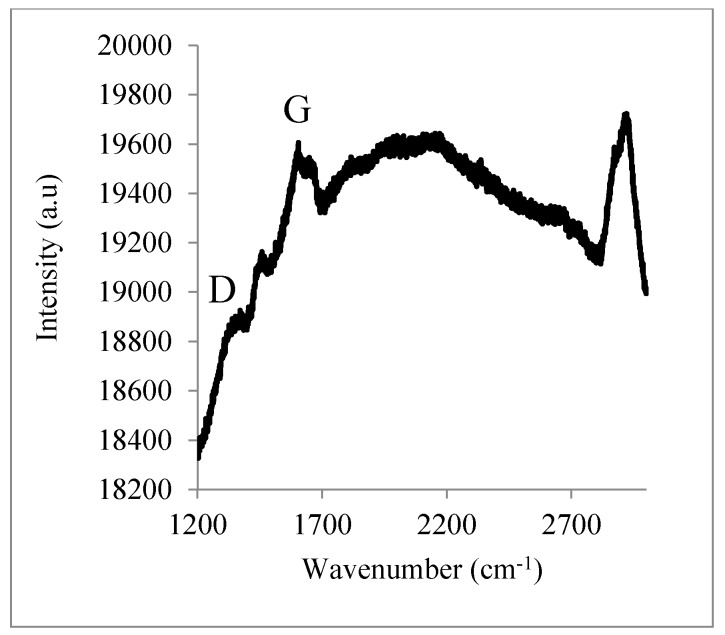
Raman spectra of the doubly clamped beam: DB501.

**Figure 12:**
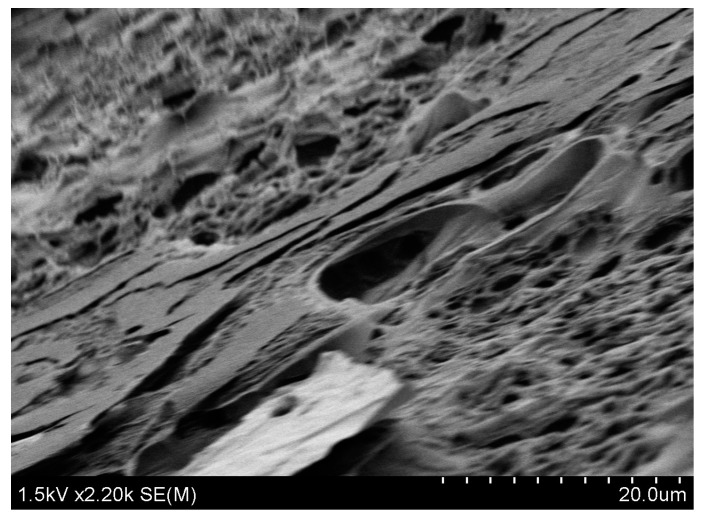
SEM image of over all of the DB150 doubly clamped beam.

**Figure 13:**
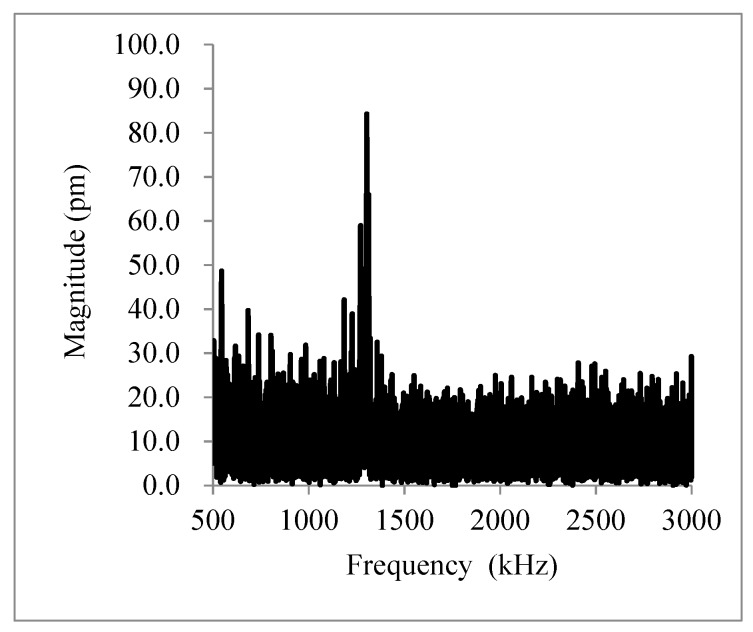
Resonance frequency of doubly clamped beam: DB150.

**Figure 14:**
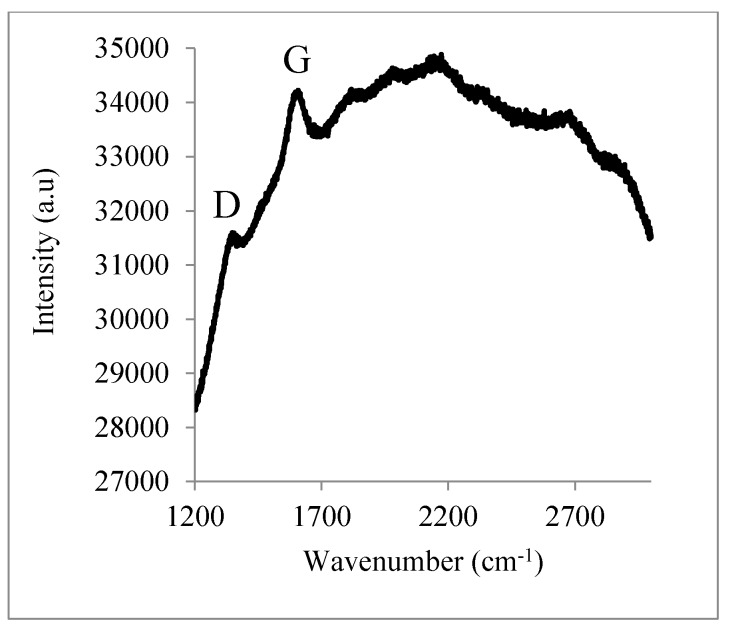
Raman spectra of the doubly clamped beam: DB150.

We also fabricated a doubly clamped beam. Figure 9, Figure 10 and Figure 11, respectively, show SEM images and clearance, frequency data, and Raman spectra for DB501. High-resolution observation was difficult because the electron beam damages carbon. Figure 9b shows the clearance between the HOPG microsheet and the resist layers. Similar results for DB150 are shown in Figure 12, Figure 13 and Figure 14.

Table 2 lists the theoretical and measured resonance frequencies. The first resonance frequency *f* is given by Equation (1). We used an *E* value of 300 GPa (HOPG) for the theoretical calculation. The ratio of measured to theoretical resonance frequencies is 0.12–0.405. DB150 has deep cracks (Figure 9 and Figure 12), suggesting that the ratio of DB150 was smaller than that of DB501. The resonance frequency of DB150 was probably affected by the nonuniform surface thickness and its numerous cracks.

The measured resonance frequencies of the cantilever were less than half of their theoretical counterparts. The authors attribute this lower resonance frequency to cracks, rough edges, and thickness variations. Such cracks, which resulted from exfoliation, generated numerous regions of small curvature. In particular, the authors believe that the beam contained numerous cracks in its interior and on its surface, which reduced the beam’s stiffness. Results of the experimental study [17] suggest that one crack reduces the resonance frequency of the cantilever by almost 5%; this value is dependent on the crack depth and location. Thus, if the beam has 15 cracks, the resonance frequency is nearly 0.4 times that of the original value. This value is dependent on the condition and number of the cracks. Thus, the ratio was consistent with our measurements of the resonance frequency. In addition, preparation of a HOPG microsheet with fewer cracks was of prime importance in our study. Defects suggested by the Raman spectra indicate the need to reconsider the patterning process, especially the O_2_ ashing step. The Raman spectra are also affected by the strong adhesion of tantalum to carbon. The difference between the theoretically calculated and the measured frequencies of cantilevers is smaller than that for double-clamped beams. The vibration amplitude of cantilever beams is shorter than that of double-clamped beams. Thus, fewer cracks occur in cantilever beams than in double-clamped beams. We infer that this is the main reason for the observed differences. The authors will thus reconsider the coating material and leave space for vibrations. Pimenta et al. studied the relation between disorder in graphite sheets and their Raman spectra [18]. The spectra of the fabricated devices show D bands. The Raman spectroscopy results imply that disorder increased as a result of our fabrication process.

Lu used bulk material (12 mm × 12 mm × 1.5 mm) cut using a wire saw and a razor blade [15]. We inferred that their plates had very few defects. Therefore, if we can prepare a 10-μm-thick graphite sheet, the vibration performance of our device can be improved. Carbonization of metal on the HOPG surface can be prevented if SiO_2_ is used as a sacrificial layer. We need to improve the etching method.

## 4. High-Precision Graphite Sheet

The typical domain size of HOPG is 1–10 µm, and its mechanical strength is as high as 300 GPa. We have some fractures and different thicknesses in the peering and polishing of the HOPG sheet. We also have damage caused by the dry and wet etching of the MEMS process. These failures degenerate the resonance frequency. We cannot control the graphite sheet thickness using our process. Therefore, it is important to develop graphite sheets with fewer domains and defects. We use a high-precision graphite sheet bonded to the MEMS base structure, with its shape formed by laser cutting. The laser focus diameter is 60 µm, the positioning resolution is 1 µm, and the X–Y travel range is 50 mm. We can therefore use laser cutting for device sizes ranging from the centimeter to the sub-millimeter scale. If micron-sized devices are needed, we will use ion-milling.

The graphite sheet is produced from aromatic polyimide film and carbonizing and graphitizing at 3000 °C in inert gas. We avoid wrinkles in the graphite sheet by applying tension and compression to the sheet during the graphitizing process. The length gauge CT6100 (HEIDENHAIN Co., Schaumburg, IL, USA) is used to measure sheet thickness.

### 4.1. Over Coating the Metal for Adhesion

**Figure 15:**
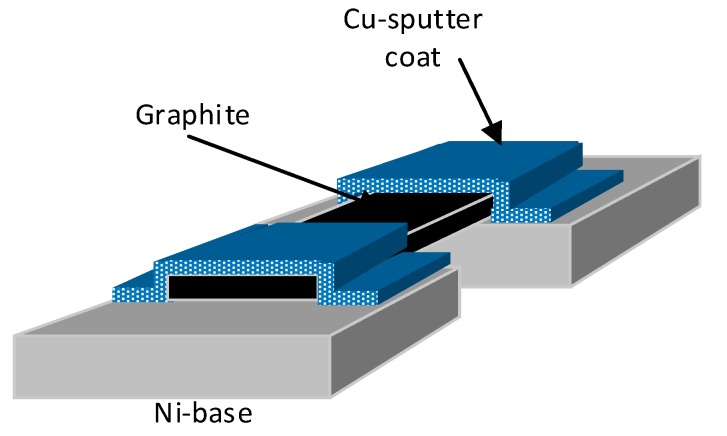
Scheme of over coating the metal method.

Figure 15 shows the scheme. We built the base platform by the electroplating of Ni. We made the base patterns easily by resist patterning of the MEMS process. We put on the graphite sheet and patterned mask. We applied a metal coating by sputter deposition in the windows of the metal patterned mask. We used a thick Cu coating approximately 2-µm thick. The Cu sputter coat of the contour lines was fixed with graphite sheet and base.

**Figure 16:**
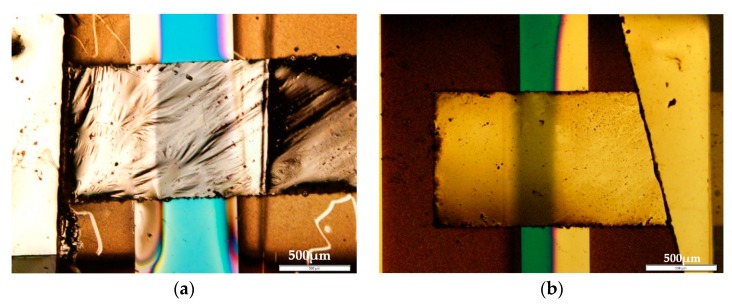
Photograph of over coating the metal method. (**a**) Case of width 1.0 mm; (**b**) Case of width 0.5 mm.

**Figure 17:**
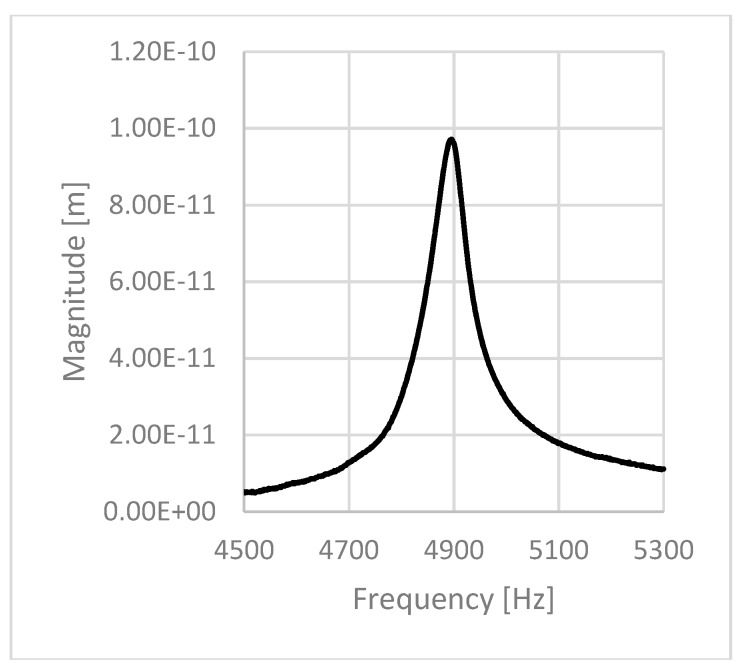
Resonance frequency of the 3.0 mm width case.

**Figure 18:**
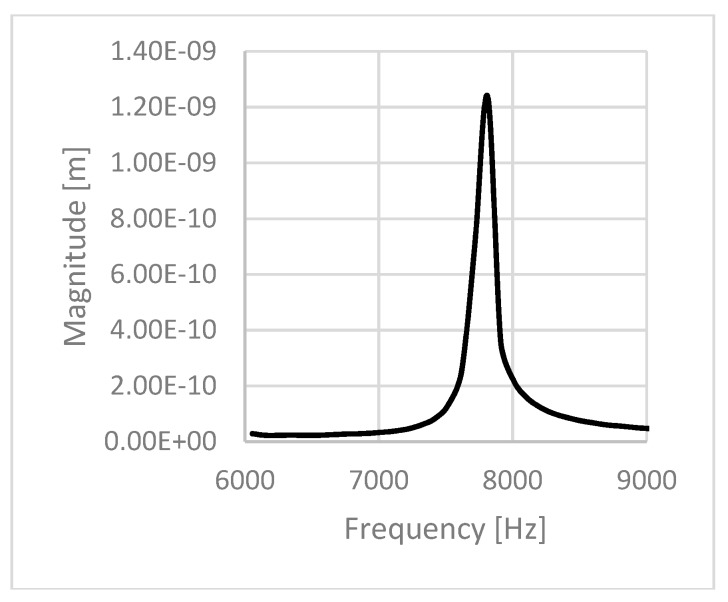
Resonance frequency of the 2.0 mm width case.

**Figure 19:**
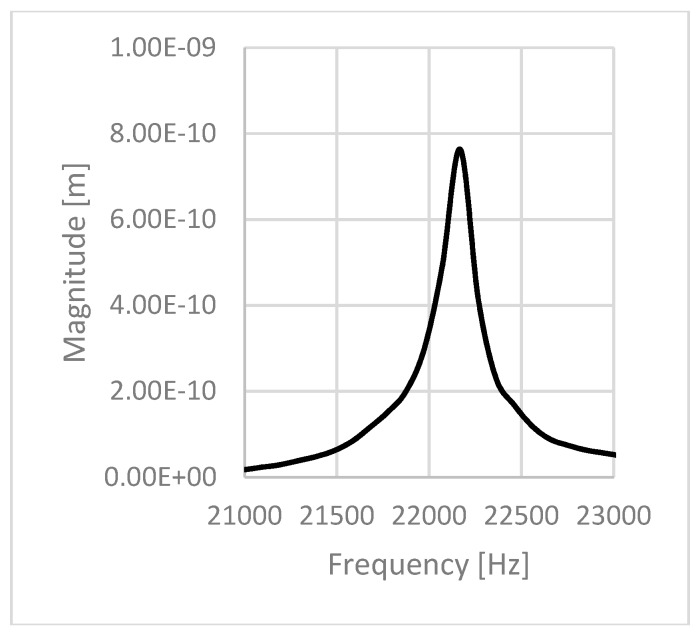
Resonance frequency of the 1.5 mm width case.

**Figure 20:**
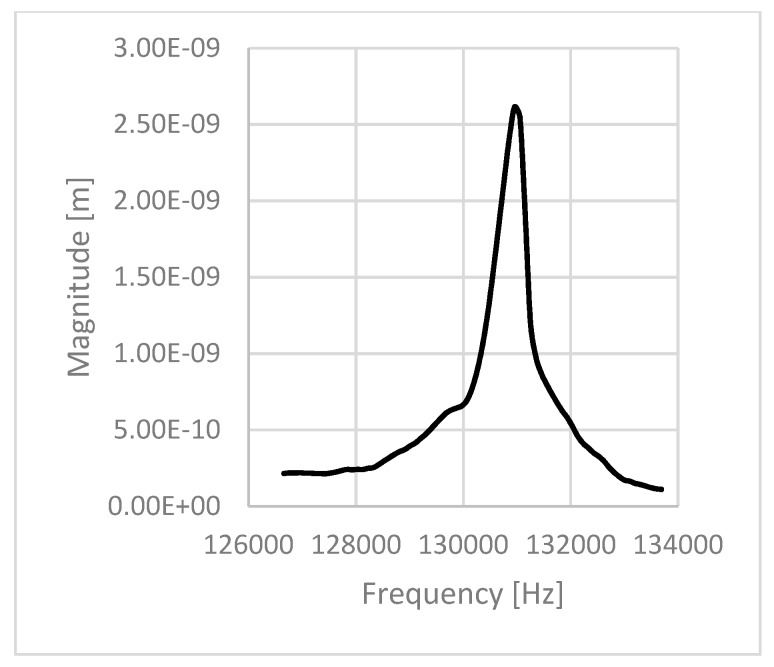
Resonance frequency of the 0.5 mm width case.

Figure 16 shows the fixed graphite sheet on the Ni base spans by Cu sputter coat. Wrinkles can be observed on the graphite sheet, and Cu sputter coating protrudes from the vibrating area of the graphite sheet caused by the clearance between the mask and the sheet. We measured the first resonance frequency under near-vacuum conditions. Figure 17, Figure 18, Figure 19 and Figure 20 show the resonance frequency results.

Table 3 lists the theoretical and measured resonance frequencies and *Q* values. We used an *E* value of 1.0 TPa for the theoretical calculation. The results show that the first resonance frequencies are almost 40–70% of the theoretical values. Quality factor values were calculated using the following equation (3 dB method):

(2) $$Q=\frac{f}{\Delta f}$$

where *f* is the peak frequency, and Δ*f* is the width of the frequency at −3 dB down from the peak. *Q* values are lower than 1000. These results are caused by a small area of adhesion, low fixing force with contour lines, and wrinkles of the graphite sheet.

### 4.2. High-Precision Graphite and Area Adhesions

**Figure 21:**
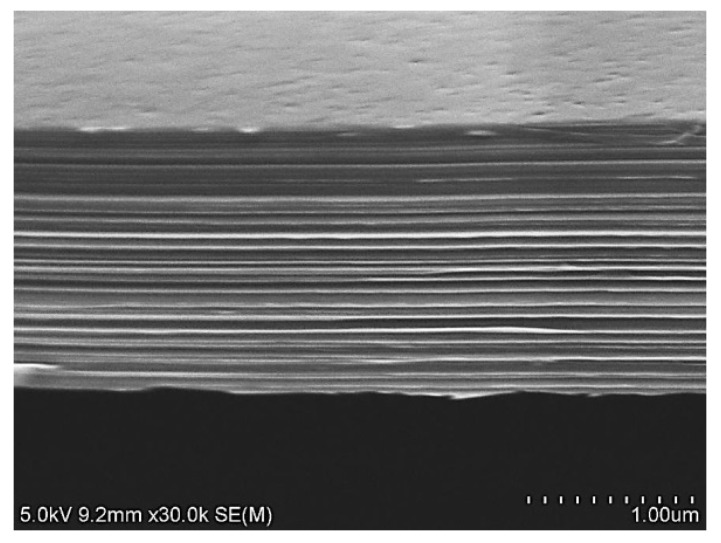
SEM images of the sheet cross-section.

**Figure 22:**
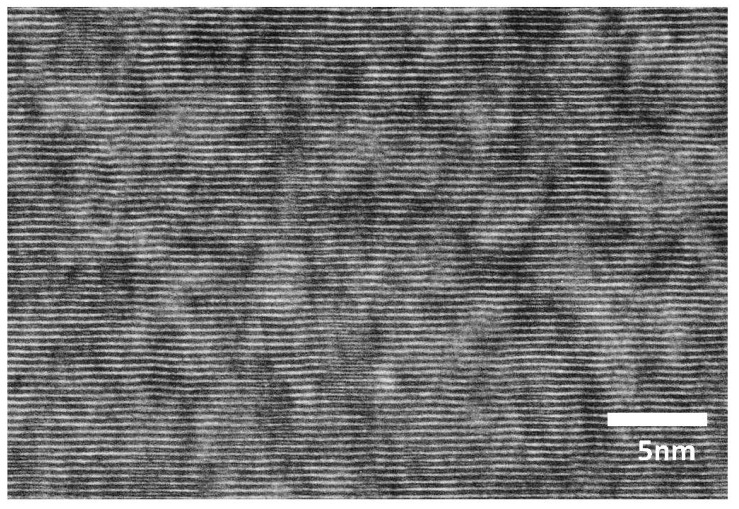
TEM images of the sheet cross-section.

A high-precision graphite sheet was produced by graphitizing a PMDA/ODA polyimide sheet at 3000 °C. This sheet is more improved from the results of Section 4.1. The thickness of the sheet is accurately controlled and tension is applied to avoid wrinkles. The surface roughness of the sheet is 0.01 µm. Figure 21 shows SEM images and Figure 22 shows TEM images of the sheet cross-section. This figure shows the highly aligned surface direction at the a–b surface. Electric conductivity is 25,300 S/cm for the 1.4-µm graphite sheet.

**Figure 23:**
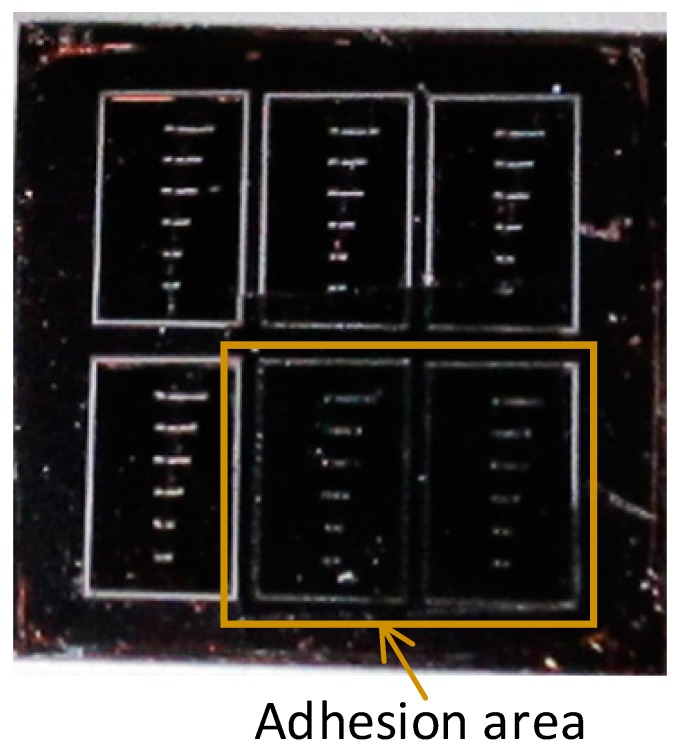
Laser cutting result of graphite sheet.

**Figure 24:**
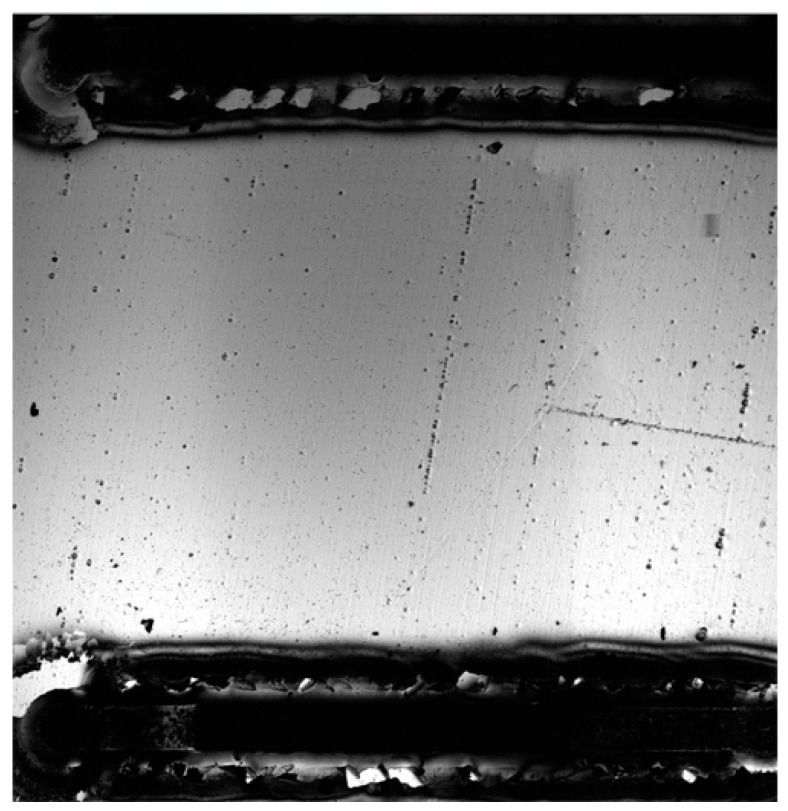
Measurment part of graphite sheet.

**Figure 25:**
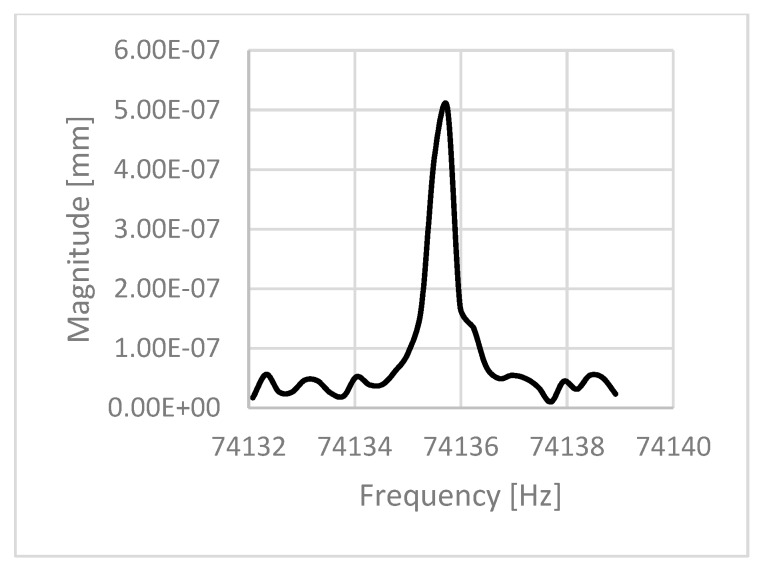
Resonance frequency of the 0.53 mm width and thickness 1.0 µm case.

**Figure 26:**
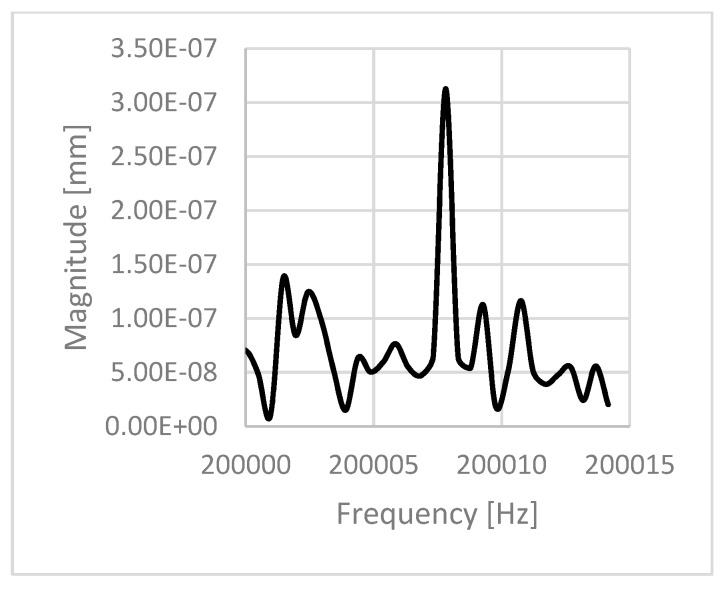
Resonance frequency of the 0.53 mm width and thickness 2.9 µm case.

**Figure 27:**
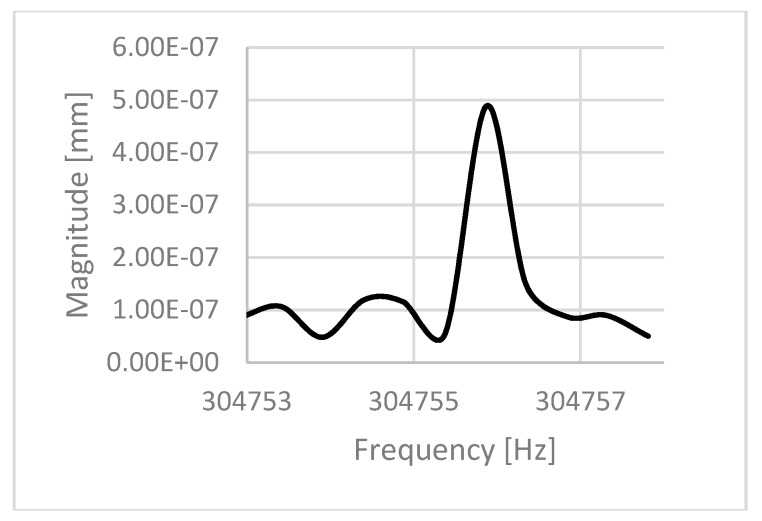
Resonance frequency of the 0.43 mm width and thickness 2.9 µm case.

The base of the double-clamped beam was trenched 30 µm by the dry etching of a Si plate. The widths of the bases are 1.03, 0.53, and 0.43 µm. The graphite sheet was fixed by spin coating a 2 µm photo resist (OFPR-800 60Cp, Tokyo Ohka Kogyo Co., LTD., Kawasaki, Japan). The photo resist was hardened by heating to 110 °C for about 3 min under a 50 g load. After fixing the graphite sheet, we use laser cutting for building the beam of the double-clamped structure. Figure 23 shows the laser cutting result. Figure 24 shows the measurement part. Figure 25, Figure 26 and Figure 27 shows the resonance frequency results.

Table 4 shows the first resonance results for the 1.0-µm-thick and 2.9-µm-thick graphite sheet. Measuring over 250 kHz is difficult owing to the method of fixing the device using tape in the chamber. From this section’s measuring of the first resonance frequency, the peak shape was sharpened and the quality factor value was improved.

These results show that the measurement values of the first resonance frequency were close to the theoretical value compared with the results of Section 4.1. Some compression stress was added by the fixing temperature of 110 °C, which caused the difference of thermal expansion rate between the Si and the graphite. *Q* values were improved to the fourth–fifth power order. Its values are 1/10 of the quarts vibrator. This improvement achieved by the wrinkleless high-precision graphite sheet and the border is formed by laser cutting after the adhesion of the sheet. We controlled the frequency simply by changing the length between clamps.

## 5. Conclusions

We used three methods for building a graphite-based MEMS vibrator. In the first trial, we used a MEMS process for building the vibrator. Avoiding cracks while using the peeling method from HOPG crystal is difficult. The formation of numerous cracks and defects and changes in the material properties lead to low performance. Thus, we used a high-accuracy graphite sheet. In the second trial, we used contour-line metal adhesion. This method could not produce sufficiently high adhesion forces. In the third trial, we used photoresist face adhesion and laser cutting for building the vibrator’s shape. Also, our graphite sheet had very few defects, cracks, and wrinkles. The vibration performance was improved 100-fold with respect to the *Q*-value. The high-accuracy graphite sheet exhibits much greater performance. Our adhesion method using a photoresist has a low adhesion force. If we can use a high-adhesion-force method, the fabrication of more precise oscillators will be possible. Metal face adhesion is a potential technique to achieve this objective. In addition, we can build a tension control mechanism for the graphite device to enable variation of the resonance frequency and compensation for temperature variance.

The fabricated vibrator is composed of simple structures and inexpensive materials, such as carbon. Our method can control the thickness easily, and we can fabricate both large and small MEMS devices. Ion milling is available for small-size cutting. Graphene is chemically stable, which enables the resonator device to be used in a wider range of applications. It also has the advantage of being applicable to MEMS devices. In addition, we can construct structural members of MEMS devices, such as mirror beams and accelerator springs. In addition, we can fabricate a millimeter-length mechanical system. We are considering additional applications of our method.

## Figures and Tables

**Table 1:** Theoretical and measurement resonance frequency of cantilevers: *b* is beam width, *L* is beam length, and *t* is beam thickness, surface indicates surface roughness.

Name	*b* (µm)	*L* (µm)	*t* (µm)	Calc. (kHz)	Meas. (kHz)	Ratio	Surface
C5	7	32	0.5	923	720	0.773	Smooth
C11	15	20	0.5	2364	1243	0.526	Smooth
C13	6	44	0.5	488	497	1.018	Rough
C19	12	15	0.5	4204	1195	0.284	Rough

**Table 2:** Theoretical and measurement resonance frequency of doubly clamped beam: *b* is beam width, *L* is beam length, and *t* is beam thickness, surface indicates surface roughness.

Name	*b* (µm)	*L* (µm)	*t* (µm)	Calc. (kHz)	Meas. (kHz)	Ratio	Surface
DB501	10	56	0.5	1899	770	0.405	Rough
DB115	5	16	0.5	23,264	545	0.023	Rough
DB150	19	24	0.5	10,339	1303	0.126	Rough
DB201	15	55	0.5	1969	780	0.396	Rough
DB202	15	26	0.5	8810	1057	0.12	Rough
DB310	8	57	1.0	3666	1313	0.358	Rough

**Table 3:** Theoretical and measured resonance frequency and *Q* value.

*L* (mm)	*t* (µm)	Calc. (Hz)	Meas. (Hz)	Ratio	*Q* Value
3.0	2.0	4832	4903	1.015	55
2.0	2.0	10,873	7813	0.719	40
1.5	2.0	19,330	22,168	1.147	123
1.0	2.0	43,493	16,756	0.385	8.9
0.5	2.5	217,465	131,054	0.603	160

**Table 4:** Theoretical and measured resonance frequency and *Q* value.

*L* (mm)	*t* (µm)	Calc. (Hz)	Meas. (Hz)	Ratio	*Q* Value
1.03	1	20,498	23,696.6	1.156	98,700
0.53	1	77,417	74,135.7	0.958	150,000
1.03	2.9	59,444	74,053.7	1.246	74,000
0.53	2.9	224,510	200,007.8	0.891	554,100
0.43	2.9	341,075	304,755.9	0.894	400,000
